# A unified framework of response surface methodology and coalescing of Firefly with random forest algorithm for enhancing nano-phytoremediation efficiency of chromium via in vitro regenerated aquatic macrophyte coontail (*Ceratophyllum demersum* L.)

**DOI:** 10.1007/s11356-024-33911-9

**Published:** 2024-06-11

**Authors:** Seyid Amjad Ali, Numan Emre Gümüş, Muhammad Aasim

**Affiliations:** 1 Department of Information Systems and Technologies, Bilkent University, Ankara, Turkey; 2 Department of Environmental Protection Technology, Kazım Karabekir Vocational School, Karamanoğlu Mehmetbey University, 70600 Karaman, Turkey; 3 Faculty of Agricultural Sciences and Technology, Sivas University of Science and Technology, Sivas, Turkey

**Keywords:** Aquatic, Artificial intelligence, Firefly algorithm, Nano-phytoremediation, Titania

## Abstract

Nano-phytoremediation is a novel green technique to remove toxic pollutants from the environment. In vitro regenerated *Ceratophyllum demersum* (L.) plants were exposed to different concentrations of chromium (Cr) and exposure times in the presence of titania nanoparticles (TiO_2_NPs). Response surface methodology was used for multiple statistical analyses like regression analysis and optimizing plots. The supplementation of NPs significantly impacted Cr in water and Cr removal (%), whereas NP × exposure time (T) statistically regulated all output parameters. The Firefly metaheuristic algorithm and the random forest (Firefly-RF) machine learning algorithms were coalesced to optimize hyperparameters, aiming to achieve the highest level of accuracy in predicted models. The *R*^2^ scores were recorded as 0.956 for Cr in water, 0.987 for Cr in the plant, 0.992 for bioconcentration factor (BCF), and 0.957 for Cr removal through the Firefly-RF model. The findings illustrated superior prediction performance from the random forest models when compared to the response surface methodology. The conclusion is drawn that metal-based nanoparticles (NPs) can effectively be utilized for nano-phytoremediation of heavy metals. This study has uncovered a promising outlook for the utilization of nanoparticles in nano-phytoremediation. This study is expected to pave the way for future research on the topic, facilitating further exploration of various nanoparticles and a thorough evaluation of their potential in aquatic ecosystems.

**Graphical Abstract:**

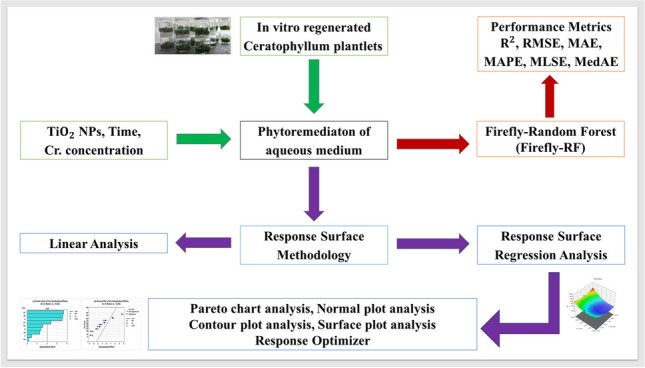

## Introduction

One of the most significant problems facing humanity today is the buildup of heavy metals in various ecological systems as a result of rapid urbanization and industrialization. These heavy metals found in soil and water are causing substantial damage like physiological or anatomical changes (Batool et al. 2015) to aquatic plants, cultivated agricultural plants, and other flora (Kumar et al. 2018). The final receptor of these pollutants are the aquatic bodies (Sadik et al. 2015), and from there, they enter into the food chain (Zheng et al. 2016). Therefore, maintaining clean aquatic environments is essential for a sustainable ecosystem. One of the major environmental risks is the discharge of Cr into the environment (Madhavi et al. 2013), and to address this grim problem, an efficient and effective approach is required, particularly in the aquatic ecosystem.

Aquatic macrophytes offer an efficient and eco-friendly approach to removing or lowering toxins in water bodies (Wei et al. 2014; Harguinteguy et al. 2016). These aquatic macrophytes absorb heavy metals from their surroundings and accumulate them in their bodies, ergo known as hyperaccumulators (Zaranyika and Nyati 2017). The process is known as phytoremediation; aquatic plants can be used for in situ phytoremediation. These plants continue to absorb metals until pollutant concentrations between plant and water bodies reach an equilibrium (Mahmood et al. 2010). *Ceratophyllum demersum* (L.) is a major aquatic macrophyte that has been effectively employed in the phytoremediation of a range of contaminants in various sources (Aasim et al. 2023a). Various studies have revealed the high potential of *C. demersum* against different heavy metals. In this regard, optimization of optimum input factors like type and concentration of pollutants, plant sample size, and culture conditions is highly significant.

Nano-phytoremediation is a relatively new bioremediation approach in the field of nanotechnology that uses plant species and biosynthesized nanoparticles to remove hazardous heavy metals from the environment. It is an effective, affordable, and environmentally responsible method (Prakash 2023). Pollutant removal from soil and water is the key advantage of using nanotechnology in phytoremediation, and both technologies operate best together (Ojuederie et al. 2022). Application of other novel technologies like nanoparticles has been reported a positive impact on phytoremediation studies (Batty and Dolan 2013). Nano-phytoremediation using different metal-based nanoparticles (NPs) against different heavy metals has been documented (Prakash 2023). The use of titania (TiO_2_) NPs in nano-phytoremediation is increasing for soil and water treatment. The phytoremediation of Cd from soil using soybean (Singh and Lee 2016) and *Brassica juncea* (Bakshi and Kumar 2023) are some examples of employing TiO_2_NPs for nano-phytoremediation. Other advantages of using TiO_2_NPs include the transformation of inorganic nitrogen into organic (Yang et al. 2006) and enhancing plant growth, development, and physiological parameters (Gao et al. 2013).

The optimization of input variables for effective phytoremediation can be attained by adopting modern statistical techniques like response surface methodology (RSM) or artificial intelligence (AI)-based modeling. RSM uses mathematical and statistical techniques to create, improve, validate, and optimize procedures and experiments (Anderson and Whitcomb 2016). This method is employed to assess the effects of discrete factors, their relative importance, the interdependence of two or more variables, and the optimal circumstances for desired experimental responses or outcomes (Farooq et al. 2013; Wantala et al. 2012). RSM is used to determine the system’s ideal operating conditions and to estimate a region that meets those conditions (Mourabet et al. 2015). The basic principle of RSM is the surface placement to comprehend the area with the most suitable response by distributing the data into maximum, local, minimum, and ridge lines. The two main experimental designs used in RSM are the Box-Behnken design (BBD) and central composite design (CCD) (Koç and Kaymak-Ertekin 2010). The experimental data are assessed for statistical model fit using the following criteria: linear, quadratic, cubic, or 2FI (two-factor interaction). Linear coefficients for independent variables are expressed as A, B, and C, whereas the interaction of inputs (AB, AC, and BC) is known as the interactive term coefficient; A^2^, B^2^, and C^2^ are quadratic term coefficients. To evaluate the model’s suitability, the correlation coefficient (*R*^2^), adjusted determination coefficient (Adj-*R*^2^), and sufficient precision are utilized. The *p* value < 0.05, lack of fit *p* value > 0.05, *R*^2^ > 0.9, and adequate precision > 4 illustrates the fitness of the model (Aydar et al. 2017). The advantages of RSM include the determination of interaction, mathematical modeling, time-saving, and extracting results with low number of trials. The fitting of experimental data to a polynomial model at the second level is the main disadvantage of RSM (Aydar 2018). The use of RSM for phytoremediation studies has been investigated in recent years using different aquatic plants against heavy metals (Ferreira et al. 2023; Kasman et al. 2019; Kumar et al. 2018; Mohamad Thani et al. 2020).

Machine learning, an advanced data analysis technique, is widely used to investigate the hidden correlations between input data and output results (Dobbelaere et al. 2021). The benefits of a multi-learning algorithm integrated model include great interpretability and good prediction performance (Zhang et al. 2022). There are several difficulties with the overall process, such as choosing an appropriate algorithm, setting up experiments, and collecting data (Bhagat et al. 2020). Artificial intelligence advancements in recent years have made it possible for researchers to estimate the removal of heavy metals using these models (Baghel et al. 2022; Shanmugaprakash et al. 2018). Numerous heavy metals research projects have used AI models and reliable models for the modeling and prediction of different heavy metal removal processes. These include the genetic algorithm (GA), multilayer perceptron (MLP), particle swarm optimization (PSO), and radial basis function (RBF) (Fan et al. 2017; Shi et al. 2023). Most recently, phytoremediation potential using different ML algorithms like multilayer perceptron and random forest (Aasim et al. 2023c) has also been documented. To the best of our knowledge, no study has been published on the use of ML models in NP-aided phytoremediation investigations. The role of TiO_2_NPs in investigating *C. demersum*’s ability to phytoremediate against Cr is presented in the current work. The novel Firefly algorithm was used to optimize the hyperparameters. The RSM statistical model was also used to examine the outcomes to optimize the input variables.

## Material and methods

### Plant material and experimental setup

The *C. demersum* plants were propagated through an in vitro regeneration protocol (Karatas et al. 2014), in the Plant Tissue Culture Lab of Sivas University of Science and Technology, Sivas, Turkiye. For phytoremediation studies, the experiments were carried out at Karamanoglu Mehmetbey University, Karaman, Turkiye. Three different input variables were used for phytoremediation studies. The TiO_2_NPs range from 1.26 to 123.74 mg/L, Cr (III) concentration of 0.684–2.317 mg/L, and the exposure time of 0.606–59. 40 h was used for designing the experiment using the design of experiment (DOE) technique. A total of 20 different combinations (runs) were extracted from DOE (Table 1) using the central composite design (CCD) of response surface methodology.

**Table 1:** Mean scores of response surface analysis of nano-phytoremediation of C*eratophyllum demersum* (L.)

Run No	TiO_2_ NP (mg/L)	Time (h)	Cr (mg/L)	Cr in Plants	Cr in Water	BCF	Cr Removal (%)
1	25.00	1.00	12.00	1256.59	0.113	1256.59	88.73
2	100.00	1.00	12.00	225.97	0.655	225.97	34.46
3	25.00	2.00	12.00	2271.73	0.383	4543.46	80.85
4	100.00	2.00	12.00	340.27	1.066	680.53	46.72
5	25.00	1.00	48.00	590.49	0.148	590.48	85.23
6	100.00	1.00	48.00	1105.47	0.420	1105.47	57.97
7	25.00	2.00	48.00	329.01	0.505	658.03	74.74
8	100.00	2.00	48.00	3901.1	0.209	7382.75	89.55
9	62.50	1.50	30.00	795.9	0.514	1193.86	65.75
10	62.50	1.50	30.00	259.30	0.725	388.95	51.69
11	62.50	1.50	30.00	281.66	0.547	422.49	63.55
12	62.50	1.50	30.00	229.63	0.537	344.45	64.22
13	1.26	1.50	30.00	2748.11	0.424	4122.17	71.76
14	123.74	1.50	30.00	205.18	0.641	307.77	57.26
15	62.50	0.68	30.00	172.90	0.296	118.18	56.65
16	62.50	2.32	30.00	2527.87	0.162	5855.81	92.99
17	62.50	1.50	0.61	229.25	0.109	343.88	92.74
18	62.50	1.50	59.39	239.90	0.562	359.85	62.52
19	62.50	1.50	30.00	264.70	0.633	397.05	57.77
20	62.50	1.50	30.00	273.12	0.705	409.68	53.02

Chromium (III) sulphate [Cr_2_(SO_4_)_3_.H_2_O] procured from (Merck®, Germany) was used as the salt for phytoremediation studies. The stock solution of Cr (III) salt was prepared (50 mg/L) using deionized water. The TiO_2_NPs (CAS: NG02MD01015; 25–45 nm, 42% wt.) were procured from nanography (ODTÜ Teknokent, Ankara, Turkiye). The stock solution of TiO_2_NPs was prepared at the rate of 10 mg/ml. Both stock solutions were stored at 4 ℃. The experiment was performed in sterilized magentas. The pH and temperature used for phytoremediation studies were performed at standard optimal protocol (Dogan et al. 2018). The aqueous solution was prepared according to the input variables [TiO_2_NPs (NP), Cr (III) concentration (*C*), and exposure time (*T*)] as given in Table 1. The 2 g/L plant samples with 5–7 cm length (Aasim et al. 2023a, 2023c) were placed in the phytoremediation mediums.

The plant samples were taken out from the phytoremediation medium after their set exposure time, followed by a waiting period of 2–3 min on the filter papers. Subsequently, samples were oven-dried for 4 days at 70 ℃. Approximately 0.5 g plant samples were digested in the microwave (CEM, MarsXpress, USA) with 6.0 ml HNO_3_ (65%) and 4.0 ml deionized water to make the final concentration of 10 ml. The dissolution conditions were set at 1600 W, 180 °C, and a waiting time of 25 min. After the drying process, filtered with Whatman filter paper and put into tubes, the final solutions were made up to 20 ml with distilled water (Dogan et al. 2018). The samples ready for analysis were measured on an inductively coupled plasma-optic absorption spectrophotometer (ICP-OES) (Agilent 720). The absorption wavelength of the measured Cr metal was Cr 205.560 nm, and a calibration curve was prepared in the range of 4.9–198 μg/L at five different concentrations. The regression coefficient was recorded in the acceptable range (*R*^2^ > 0.999). The UME CRM 1201 reference material was used to calculate the limit of detection (LOD), limit of quantification (LOQ), and precision values (Table 2). The analytical method validation of the ICP-OES was performed by the Eurachem guideline (EURACHEM, 1998). The Cr contents in plants (mg/kg dry weight) were measured by using an appropriate conversion formula, whereas Eqs. 1 and 2 were employed for BCF of plants (Dogan et al. 2018; Zayed et al. 1998) and Cr removal (%).

**Table 2:** Linear range, regression, correlation coefficient (R^2^), LOD, LOQ, and precision (RSD %) scores for Cr analysis

Liner range (μg/L)	4.9–198
Regression	$\text{y}=1.8\text{x}+14.6$
Correlation coefficient (R^2^)	0.999
Precision (RSD %)	5.412
LOD (μg/L)	7.154
LOQ (μg/L)	23.379

1 $$\text{BCF}= \frac{\text{Trace element concentration in plant tissue }(\text{mg}/\text{kg})}{\text{First concentration of the element added to deionized water }(\text{mg}/\text{L})}$$

2 $$\text{Cr removal}\left(\%\right)=\frac{\text{Initial Cr concentration}-\text{Cr concentration after phytoremediation}}{\text{Initial Cr concentration}}\times 100$$

### Response surface analysis

The phytoremediation potential and optimization of Cr removal from aqueous solution using in vitro propagated aquatic macrophyte (*C. demersum*) with the aid of RSM were investigated in this study. The design matrix of three input variables [TiO_2_NPs (NP), concentration (*C*), and exposure time (*T*)] using central composite design was generated with a total of 20 combinations with six common points. The results were analyzed for individual input variables and interactions of two input variables (NP × *C*, NP × *T*, *C* × *T*). The results were computed and analyzed by examining the overall model analysis and constructing different plots to check the impact and relationship between input variables, contour and surface plots, and response optimizer. The regression analysis, Pareto charts, normal plots, and response optimization were performed with Minitab. Expert design program was used for constructing contour and surface plots.

### Machine learning analysis

Machine learning algorithms necessitate hyperparameter optimization by either grid or random search to find the complicated and nonlinear behaviors of predictive and predicted components. Grid search is time-exhaustive and sluggish since it attempts all potential parameter combinations to get the best hyperparameters. Random search, on the other hand, employs a certain number of random parameter possibilities to discover the best combination of parameters. Although random search is considerably faster than grid search, it is unlikely to get the optimal combination of parameters as it may not converge to a global optimum. In recent years, more powerful hyperparameters optimizing algorithms have been documented like Firefly algorithms. Based on the flashing behavior of fireflies, the Firefly algorithm is a multimodal metaheuristic algorithm that draws inspiration from nature to find either potential mates or pray (Moazenzadeh et al. 2018; Yang 2009). Fireflies employ a chemical process to produce bioluminescence to emit tiny, distinct rhythmic flashes that attract one another (Moazenzadeh et al. 2018). The Firefly algorithm has two benefits over other similar algorithms. It is first and foremost an attraction-based system, and appeal declines with distance. This implies that the entire population is automatically split into smaller groups that gather around nearby optima until the optimum option is identified. Furthermore, because of these subgroups, the Firefly algorithm can simultaneously discover all optimum modes (Yang and He 2013). This optimization algorithm computes the brightness of a Firefly in the backdrop of the objective function. The amount of attraction and brightness between two fireflies is determined by the separation distance between them (Nayak et al. 2016).

**Fig. 1:**
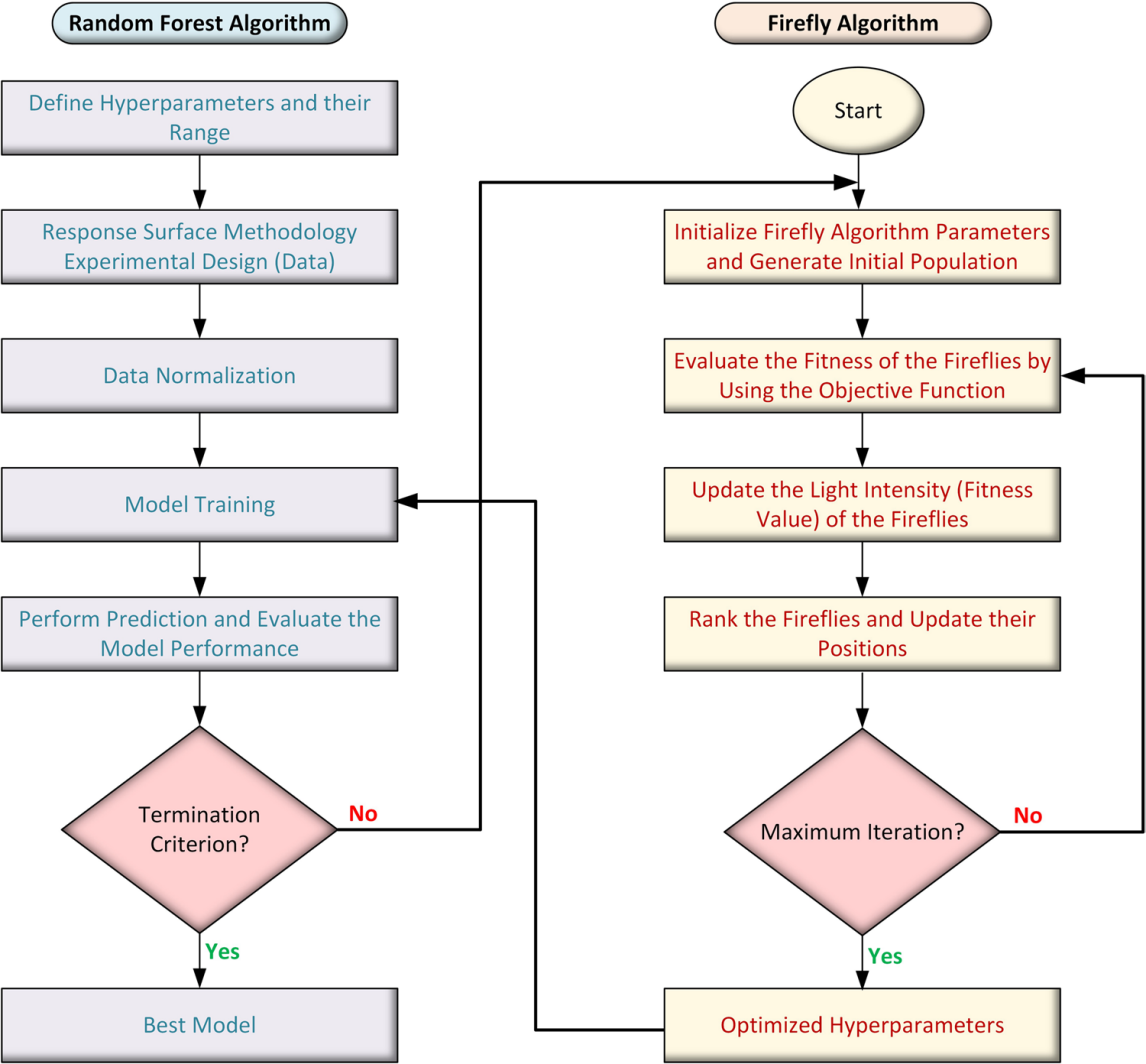
An overview of the working principle of the Firefly algorithm to optimize hyperparameters for the random forest algorithm

In this work, Python programming language (Van Rossum and Drake 2009) was used to implement a custom code to implement the random forest (RF) machine learning algorithm together with the sklearn-nature-inspired algorithms package^1^ for hyperparameter tuning. This enabled us to benefit from both the prediction capabilities of RF and the optimization potential of Firefly algorithms to get the best results (Fig. 1). Random forest is one of the most popular advanced decision tree models in data science (Aggarwal 2018), which trains several trees simultaneously using bagging (bootstrap aggregation). Equation 3 presents the fundamental concept of the entire operating mechanism, and almost all trained trees have an impact on the outcome (Pavlov 2019).

3 $$y=\sum_{i=1}^{n}({\alpha }_{i}-{\alpha }_{i}^{*})k\left(x,{x}_{i}\right)+b$$

*y* = data point value; *n* = sampling size (number).

Moreover, the customized code made use of the leave-one-out cross-validation (LOO-CV) technique to predict the results and assess the model’s efficacy (Webb et al. 2011). Equations 4–10 employed six different performance measures to assess the model’s usefulness.

4 $${R}^{2}=1- \frac{{\sum }_{i=1}^{n}{({Y}_{i}-{\widehat{Y}}_{i})}^{2}}{{\sum }_{i=1}^{n}{({Y}_{i}-\widetilde{Y})}^{2}}$$

5 $$\text{RMSE}= \sqrt{\frac{1}{n} {\sum }_{i=1}^{n}{({Y}_{i}-{\widehat{Y}}_{i})}^{2}}$$

6 $$\text{RRMSE} (\%)= \sqrt{\frac{1}{n}\frac{{\sum }_{i=1}^{n}{({Y}_{i}-{\widehat{Y}}_{i})}^{2}}{{\sum }_{i=1}^{n}{\widehat{Y}}_{i}^{2}}}\times 100$$

7 $$\text{MAE}= \frac{1}{n} \sum_{i=1}^{n}\left|{Y}_{i}-{\widehat{Y}}_{i}\right|$$

8 $$\text{MAPE}= \frac{1}{n} \sum_{i=1}^{n}\left|\frac{{Y}_{i}-{\widehat{Y}}_{i}}{{Y}_{i}}\right|\times 100$$

9 $$\text{MSLE}= \frac{1}{n} \sum_{i=1}^{n}{\left(\text{log}\left({Y}_{i}+1\right)-\text{log}\left({\widehat{Y}}_{i}+1\right)\right)}^{2}$$

10 $$\text{MedAE}= \text{median}\left(\left|{Y}_{1}-{\widehat{Y}}_{1}\right|,\dots ,\left|{Y}_{n}-{\widehat{Y}}_{n}\right|\right)$$

*R*^2^: The coefficient of determination, which ranges from 0 to 1, analyzes a machine learning model’s predictive capability for an outcome. It assesses how successfully a statistical model predicts an outcome, with values closer to 1 indicating higher projected accuracy.
RMSE: Root mean squared error is a commonly used metric for determining the accuracy of predictive models. It measures the differences between predicted and actual values by squaring the errors, averaging them, and then calculating the square root. It provides insight into the model’s performance, with lower values indicating more predicted accuracy.
RRMSE (%): Relative root mean squared error (%) assesses predictive model accuracy in relation to the target variable’s range of values. It normalizes RMSE according to the target variable range and displays it as a percentage for easier comparison across datasets or variables. Smaller values indicate better performance of the model.
MAE: Mean absolute error is also frequently denoted as L1 loss. It is notable for being among the most straightforward and easily understood loss functions and assessment measures. All of the dataset’s absolute differences between predicted and actual values are averaged to determine its value. It is simply the average of absolute errors, focusing only on their size, and independent of direction. Better model accuracy is indicated by lower MAE values.
MAPE: Mean absolute percentage error is calculated by dividing the absolute difference between the actual and predicted values by the actual value, yielding an absolute percentage. The values are then averaged over the dataset. MAPE grows proportionately to error, with lower values suggesting better model performance. A model is considered worthwhile only if its MAPE falls below 50%.
MSLE: Mean squared logarithmic error is a measure used to evaluate the accuracy of a forecasting model, particularly when the data has a wide range of values. It measures the average of the squared differences between the logarithms of the predicted and actual values.
MedAE: Median absolute error is primarily interesting since it can resist outliers. This loss is calculated by taking the median of all absolute discrepancies between the actual and predicted values. Its best possible score is 0, with lower values indicating higher performance.

Prior to model training and testing, all input features underwent standardization, where their values were scaled to be centered on a mean of zero and retain a unit standard deviation by using the formula of Eq. 11. This transformation enables the inputs to be dimensionless or comparable, thereby enhancing both algorithm performance and data quality.

11 $${X}{\prime}=\frac{{X}_{i}-\mu }{\sigma }$$

${X}{\prime}$ is the standardized value, ${X}_{i}$ is the actual data, $\mu$ is the mean of the feature values, and $\sigma$ is the standard deviation of the feature values.

## Results

### Response surface model analysis

Response surface regression indicated that all output parameters had a statistically significant influence, resulting in a significant model (Table 3). The scores of *R*^*2*^-Sq (act) and *R*^*2*^ (pred) values of all output variables followed the order from maximum to minimum as BCF (*R*^*2*^_act_ 78.08%, *R*^*2*^_pred_ 51.93%) > Cr uptake by plants (*R*^*2*^_act_ 75.95%, *R*^*2*^_pred_ 48.31%) > Cr removal % (*R*^*2*^_act_ 70.01%, *R*^*2*^_pred_ 39.23%) > Cr in water (*R*^*2*^_act_ 62.41%, *R*^*2*^_pred_ 22.42%). The *R*^*2*^ (pred) scores were relatively less than *R*^*2*^ (act) irrespective of output parameters. The regression equations of all parameters were recorded as follows

**Table 3:** Statistical analysis of response surface regression model for phytoremediation of Cr

Input	Cr in Plants	Cr in Water	BCF	Cr Removal (%)
Model	0.000**	0.000**	0.000**	0.000**
NPs (NP)	0.059	0.001**	0.190	0.000**
Concentration (C)	0.000**	0.177	0.000**	0.003**
Time (T)	0.250	0.661	0.299	0.781
NP × C	0.081	0.218	0.142	0.005**
NP × T	0.000**	0.001**	0.000**	0.001**
C × T	0.249	0.127	0.255	0.421
**R**^**2**^** act. (%)**	75.95	62.41	78.08	70.01
**R**^**2**^** adj. (%)**	68.74	51.13	71.50	61.02
**R**^**2**^** pred. (%)**	48.31	22.42	51.93	39.23

12 $$\varvec{Cr\; uptake\; by\; plants}=8397-107.4 NP-5267 Cr-101.4 time+0.3246 NP\times NP+1636 Cr\times Cr-0.029 time\times time+14.38 NP\times Cr+1.305 NP\times time+19.5 Cr\times time$$

13 $$\varvec{Cr\; in\; water}=2.047+0.01483 NP+1.922 Cr+0.0395 time-0.000004 NP\times NP-0.477 Cr\times Cr-0.000245 time\times time-0.00286 NP\times Cr-0.000231 NP\times time-0.00742 Cr\times time$$

14 $${\varvec{BCF}}=14213-166.0 NP-10754 Cr-171.5 time+0.457 NP\times NP+3727 Cr\times Cr-0.174 time\times time+22.5 NP\times Cr+2.247 NP\times time+36.2 Cr\times time$$

15 $$\varvec{Cr\; removal\; (\%)} =193.6-1.380 NP-78.3 Cr-2.289 time+0.00070 NP \times NP+19.39 Cr \times Cr+0.01821 time \times time+0.415 NP \times Cr+0.01406 NP \times time+0.232 Cr \times time$$

Investigating individual input variables showed the insignificant impact of time on all output parameters, whereas the variable impact of NPs and concentration on output parameters were recorded. The statistically significant impact of NPs on Cr (water) and Cr removal (%) was documented. Computation of concentration revealed an insignificant impact on Cr (water) and a statistically significant impact on remaining output parameters respectively. Analysis of interaction exhibited the statistically significant impact of NP × *T* and insignificant impact of *C* × *T* for all output parameters, whereas Cr removal (%) was statistically significant from NP × *C* interaction. The means of all output variables in response to all input variables are presented in Table 1. Results demonstrated the significant relationship between input variables and output parameters. Analysis of phytoremediation studies revealed the maximum Cr removal from run 22 (92.74%), followed closely by run 17 (92.74%), and run 8 (89.55%).

### Pareto and normal plot analysis

**Fig. 2:**
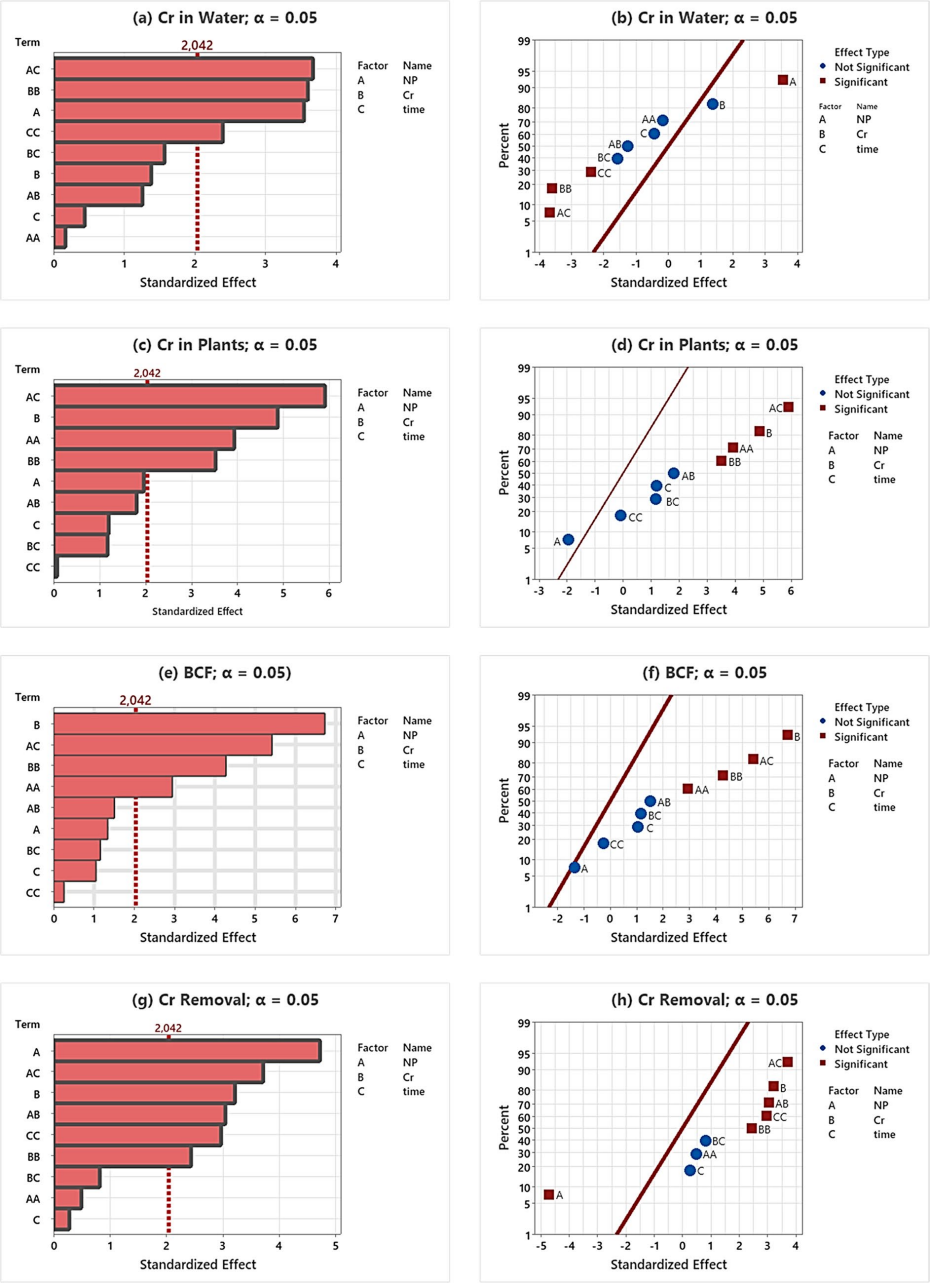
Pareto chart and normal plot-based analysis of nano-phytoremediation

The standardized effects of all individual and interactive effects of input variables on phytoremediation variables were illustrated by employing the Pareto chart and normal plots (Fig. 2a–h). The value of the Pareto chart was recorded as 2.042 (Fig. 2a, c, e, f) and all input variables into significant and insignificant variables. The standardized values equal to or above the 2.042 scores were statistically significant (expressed as bold) and vice versa. Results revealed the ranking of significant levels of **AC** > **BB** > **A** > **CC** > BC > B > AB > C > AA for Cr in water (Fig. 2a), **AC** > **B** > **AA** > **BB** > A > AB > C > BC > CC for Cr in plants (Fig. 2c), **B** > **AC** > **BB** > **AA** > AB > A > BC > C > CC for Cr in plants (Fig. 2e), and **A** > **AC** > **B** > **AB** > **CC** > **BB** > BC > AA > C for Cr removal (Fig. 2g).

The significance level of input variables was also checked by normal plots for all output variables. The distribution of input variables on the right side of the red line exhibits a positive correlation and vice versa negative correlation for variables located on the left side, whereas red square and blue-circled symbols illustrate the significant and insignificant impact, respectively. Another significance of the normal plots includes the positioning of the input variables from top to bottom based on their significance level. The input variables positioned on the top of the table reflect the more significant level and vice versa decreased with descending pattern. Results of normal plots for Cr in water displayed the statistically significant and positive correlation of NP concentration (red square), whereas a statistically significant and positive correlation was scored for NP × time factors. The interaction of NP × Cr and Cr × *T* was recorded as statistically insignificant and negative. Both Cr concentration and time factor were also statistically insignificant but remained positive and negative, respectively (Fig. 2b). Results revealed that irrespective of NP concentration, all other input variables reflected the positive correlation for Cr in plants and Cr in plants, irrespective of them being statistically significant or insignificant (Fig. 2d, 1h). Figure 2d also depicted that Cr concentration (*C*) and Cr × *T* expressed a positive impact on Cr concentration in plants. However, statistically significant and positive correlations were computed for NP × *T*, NP × Cr, and Cr concentration (Fig. 2h). Figure 2f illustrates the positive correlation between all input factors and Cr in plants. However, statistically significant and positive correlations were scored for Cr concentration and NP × *T*. Statistically insignificant and positive correlations were observed for individual NP and Cr concentration, and NP × Cr and Cr × *T* factors (Fig. 2f). It is evident from the results that NP concentration affected the phytoremediation of Cr by *C. demersum* plants.

### Contour and surface plot analysis

**Fig. 3:**
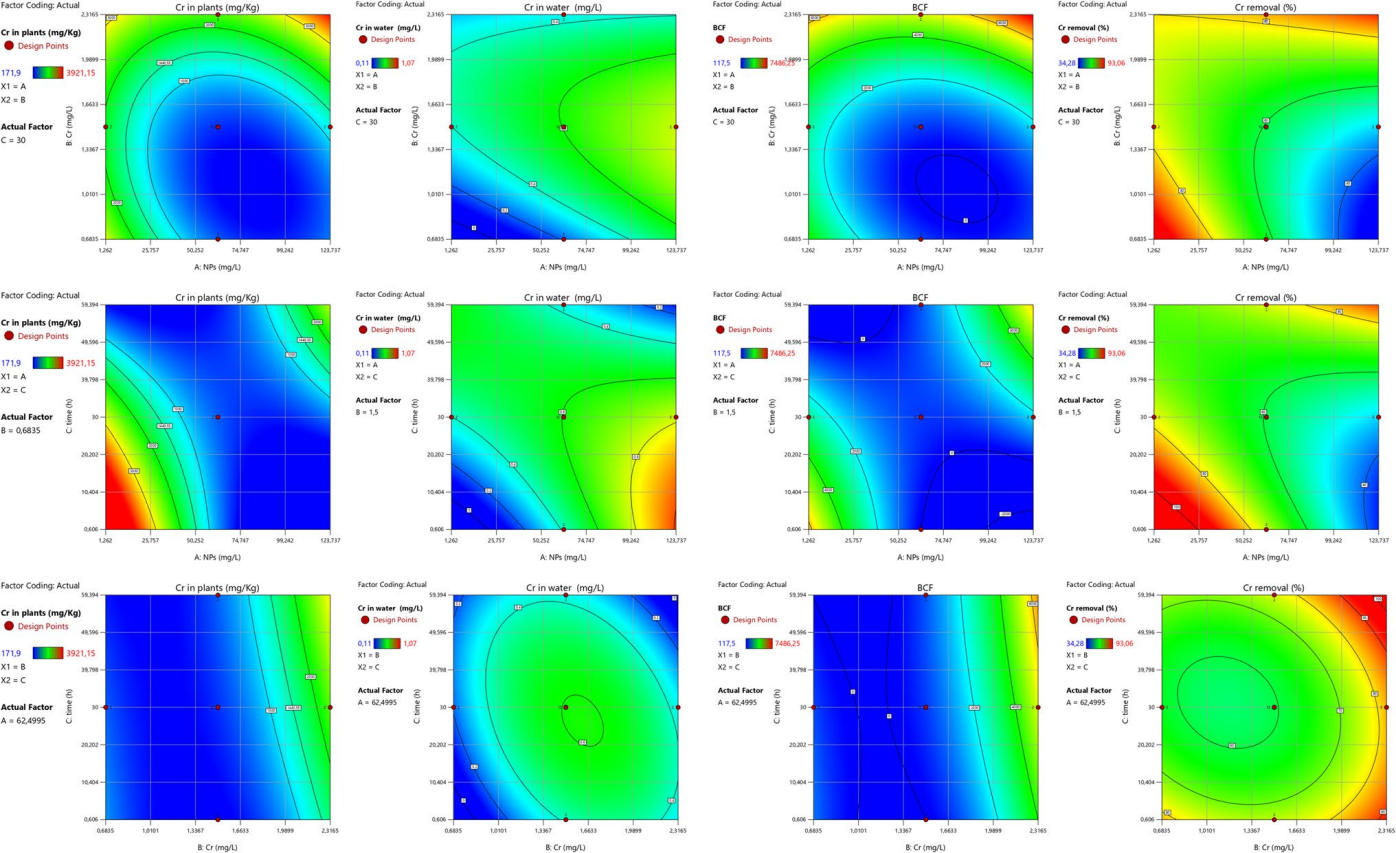
2D contour plots for nano-phytoremediation of Cr

**Fig. 4:**
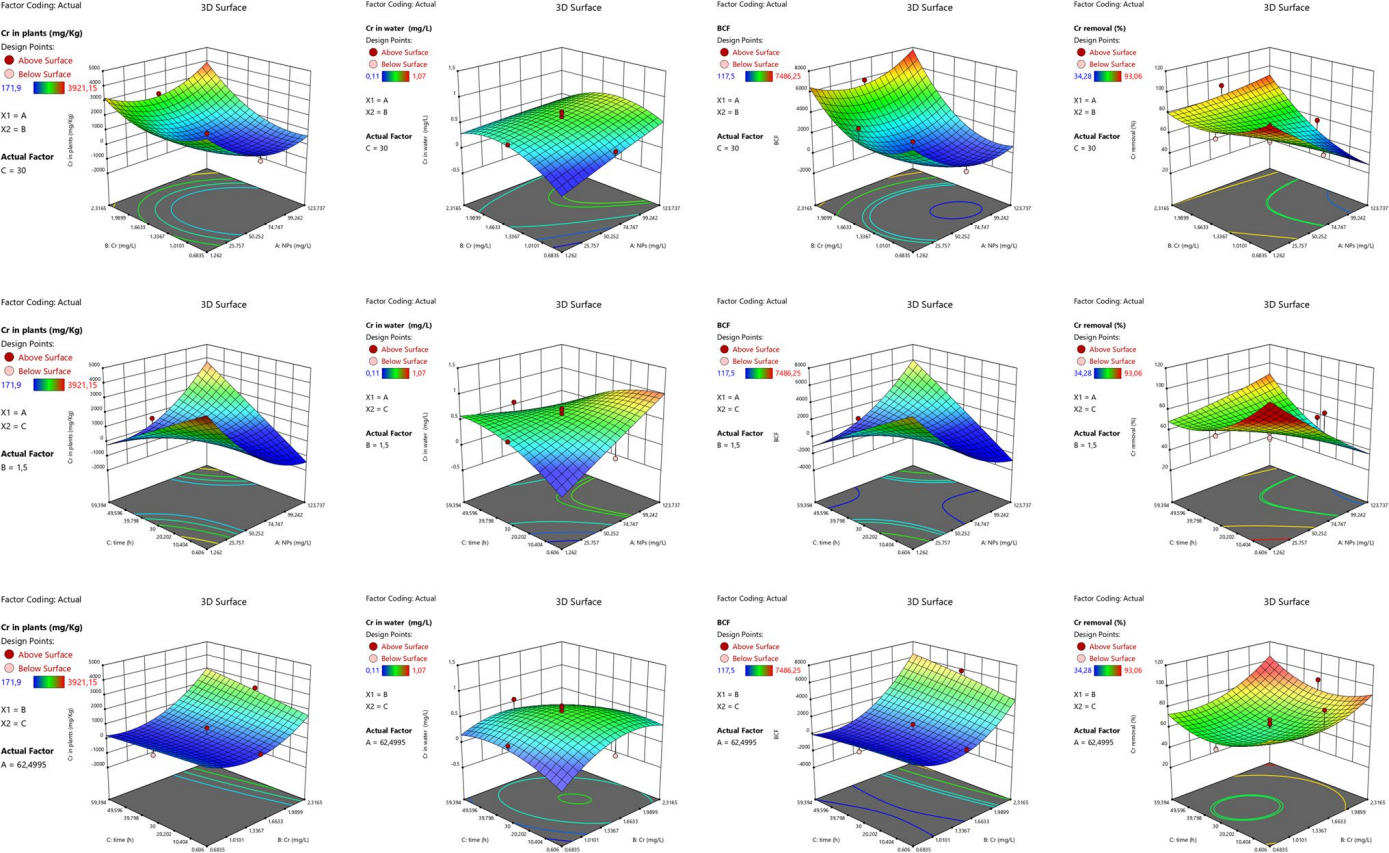
3D surface plots for nano-phytoremediation of Cr

The results of phytoremediation studies were analyzed by computing 2D contour plots (Fig. 3a–d) and 3D surface plots (Fig. 4a–d) for NP × C, NP × *T*, and *C* × *T* interactions for all output parameters used for phytoremediation studies. The interaction of NP × *C* and NP × *T* revealed the Cr concentration in plants over 3000 mg/kg and 2000 mg/kg for *C* × *T* interaction (Fig. 3a–c). Investigating the contour plots of all possible interactions revealed the chance of zero Cr in the solution (Fig. 3d–f). Considering the BCF values, the interaction of NP × *C* yielded the possible BCF values in the range of 6000–7500, whereas *N* × *T* and *C* × *T* provided BCF values of slightly over 6000 and more than 6000, respectively (Fig. 3g–i). The Cr removal (%) was optimized between 80 and 100% with very low chances of 100% from NP × *C*, whereas the remaining interactions provided the chance of 100% Cr removal (Fig. 3j–l). A similar pattern of interactions of two input variables on their respective output variables was observed in surface plots (Fig. 4a–l).

### Response optimizer analysis

A comparison of the results of actual and predicted values presented the best combination from runs 8, 16, and 17. The results were used to optimize the input variables by setting the individual target of all output variables (Table 4). The combined optimized values of all input variables with fitted values of all output parameters are also presented in Table 5. Results revealed the best combination of 123.737 mg/L NP, 2.32 mg/L Cr, and 59.39 h time for the set target based on maximum and minimum. The system was also used for a desired target of 100% Cr removal (Table 4). Results revealed the combination of 42.25 mg/L NP, 0.684 mg/L Cr, and 0.61 h time for 100% removal of Cr from the aqueous medium (Table 5).

**Table 4:** Multiple response prediction of set target for all output variables

Response	Target	Lower	Target	Upper	Weight	Importance
Cr Removal (%)	Maximum	34.281	93.06	-	1	1
BCF	Maximum	117.500	7486.25	-	1	1
Cr in Water	Minimum	-	0.11	1.067	1	1
Cr in Plants	Maximum	171.904	3921.15	-	1	1
Response		Fit	SE Fit	95% CI	95% PI	
Cr Removal (%)		138.5	15.1	(107.7; 169.4)	(101.3; 175.8)	
BCF		12890	1654	(9512; 16268)	(8810; 16970)	
Cr in Water		-0.436	0.251	(-0.949; 0.076)	(-1.056; 0.183)	
Cr in Plants		6937	881	(5137; 8737)	(4762; 9112)	
Optimized Input Variable	Setting					
NP	123.74					
Cr	2.32					
Time	59.39

**Table 5:** Multiple response prediction of set target for Cr removal

Response	Goal	Lower	Target	Upper	Weight		Importance
Cr Removal (%)	Target	34.2812	100	110	1		1
Response		Fit	SE Fit	95% CI		95% PI	
Cr Removal (%)		100.0	10.6	(78.4; 121.6)		(69.9; 130.1)	
Optimized Input Variable		Setting					
NP		45.25					
Cr		0.684					
Time		0.61					

### Heatmap analysis

**Fig. 5:**
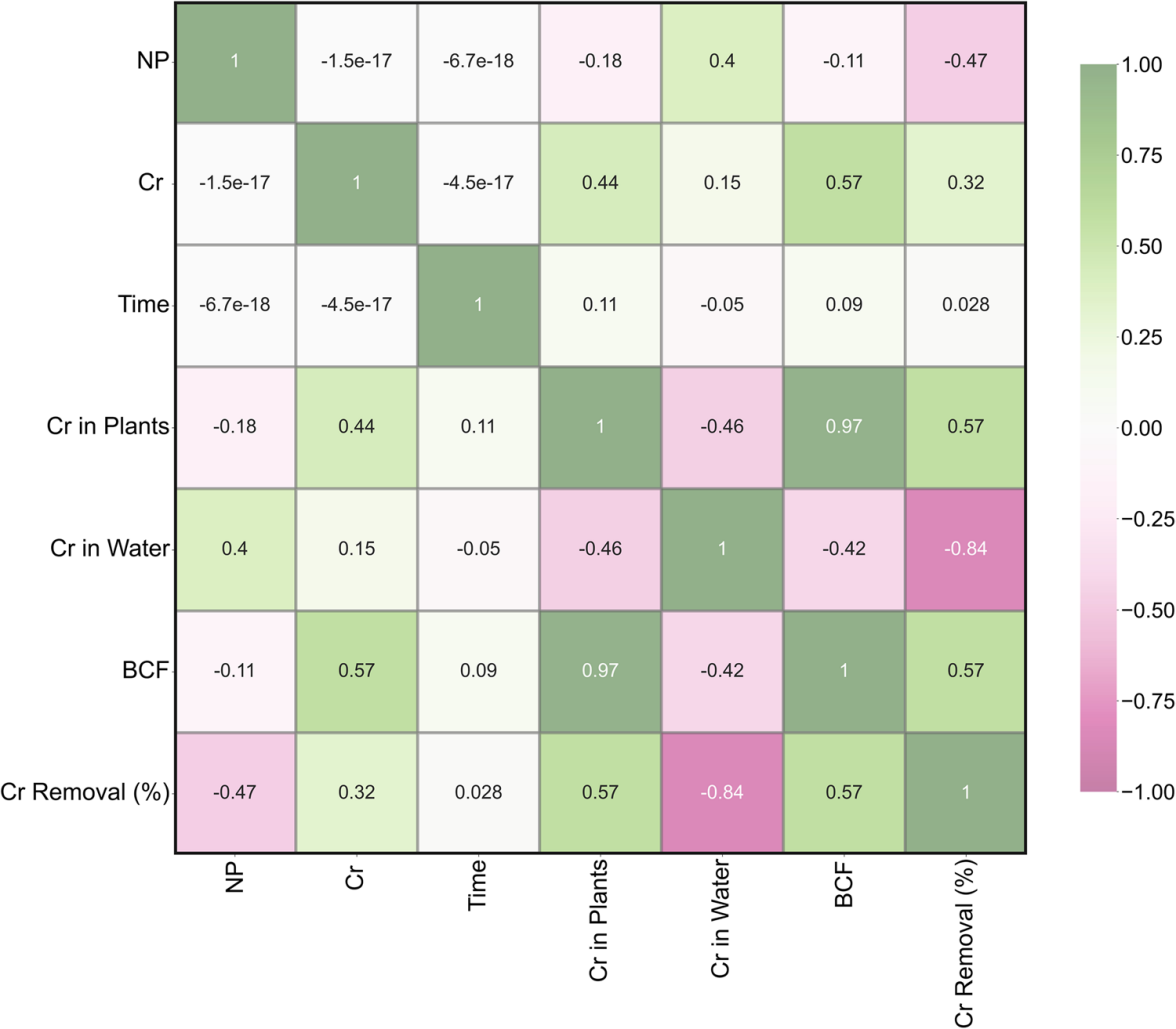
Correlation heatmap between input and output variables for nano-phytoremediation of Cr

To identify potential correlations between the variables and evaluate the strength of these connections, a heatmap correlation analysis was performed for each of the input and output variables. Figure 5 visually represents the correlation between variables on each axis through individual squares. The concentration of Cr in water exhibited a weak negative correlation (− 0.05) with time, a low positive correlation (0.15) with Cr concentration, and a positive correlation (0.4) with NPs. For Cr in plants, there was a weak positive connection (0.11) with time, a medium positive correlation (0.44) with Cr concentration, and a low negative correlation (− 0.18) with NPs. Regarding BCF, a weak positive connection (0.09) with time, a medium positive correlation (0.57) with Cr concentration, and a weak negative correlation (− 0.11) with NPs were identified. Finally, Cr removal (%) displayed a weak positive connection with time (0.028), a medium positive correlation with Cr concentration (0.32), and a medium negative correlation (− 0.47) with NPs.

### Machine learning analysis

**Fig. 6:**
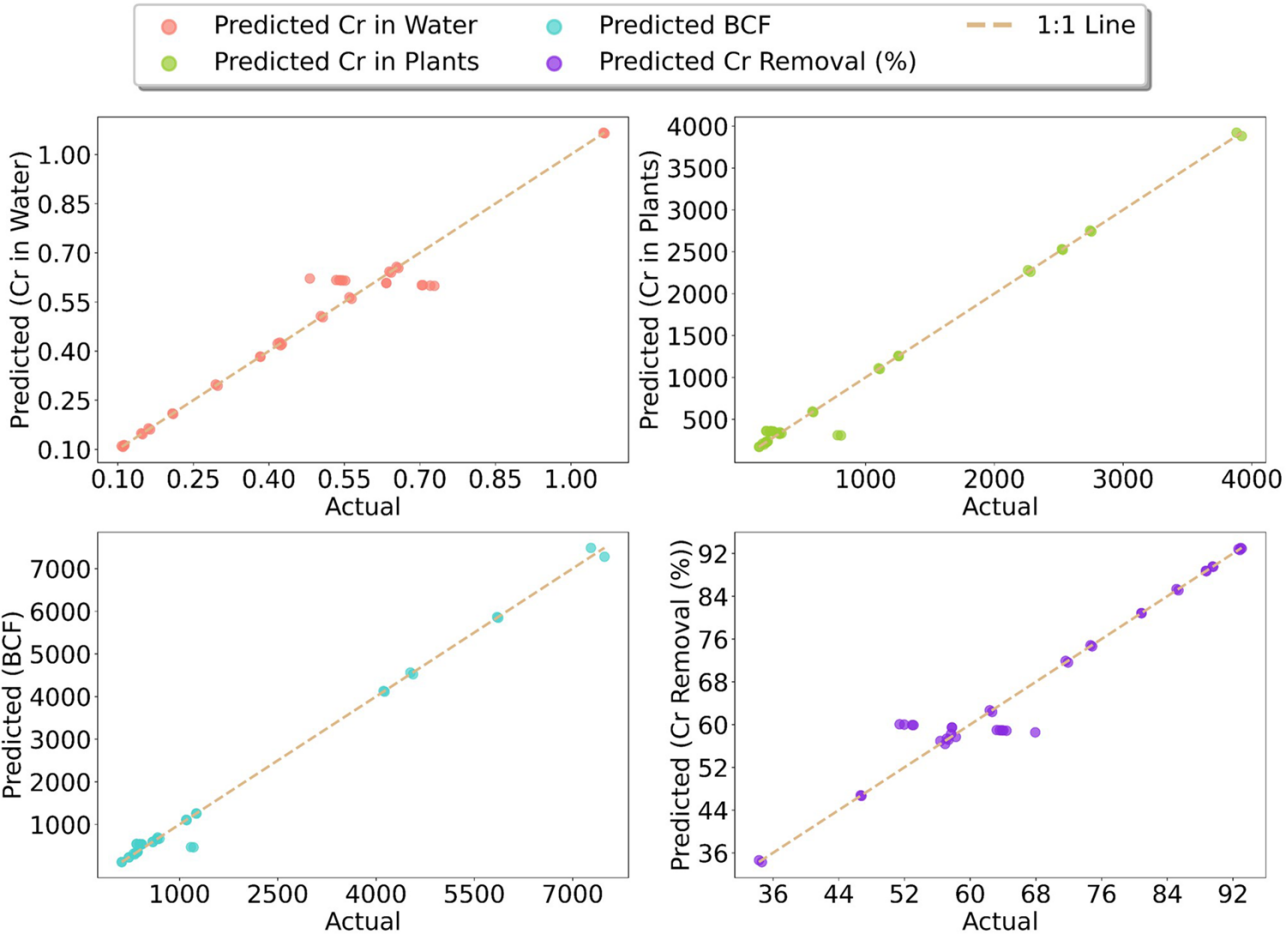
Actual and predicted scores of nano-phytoremediation via Firefly-RF algorithm

**Fig. 7:**
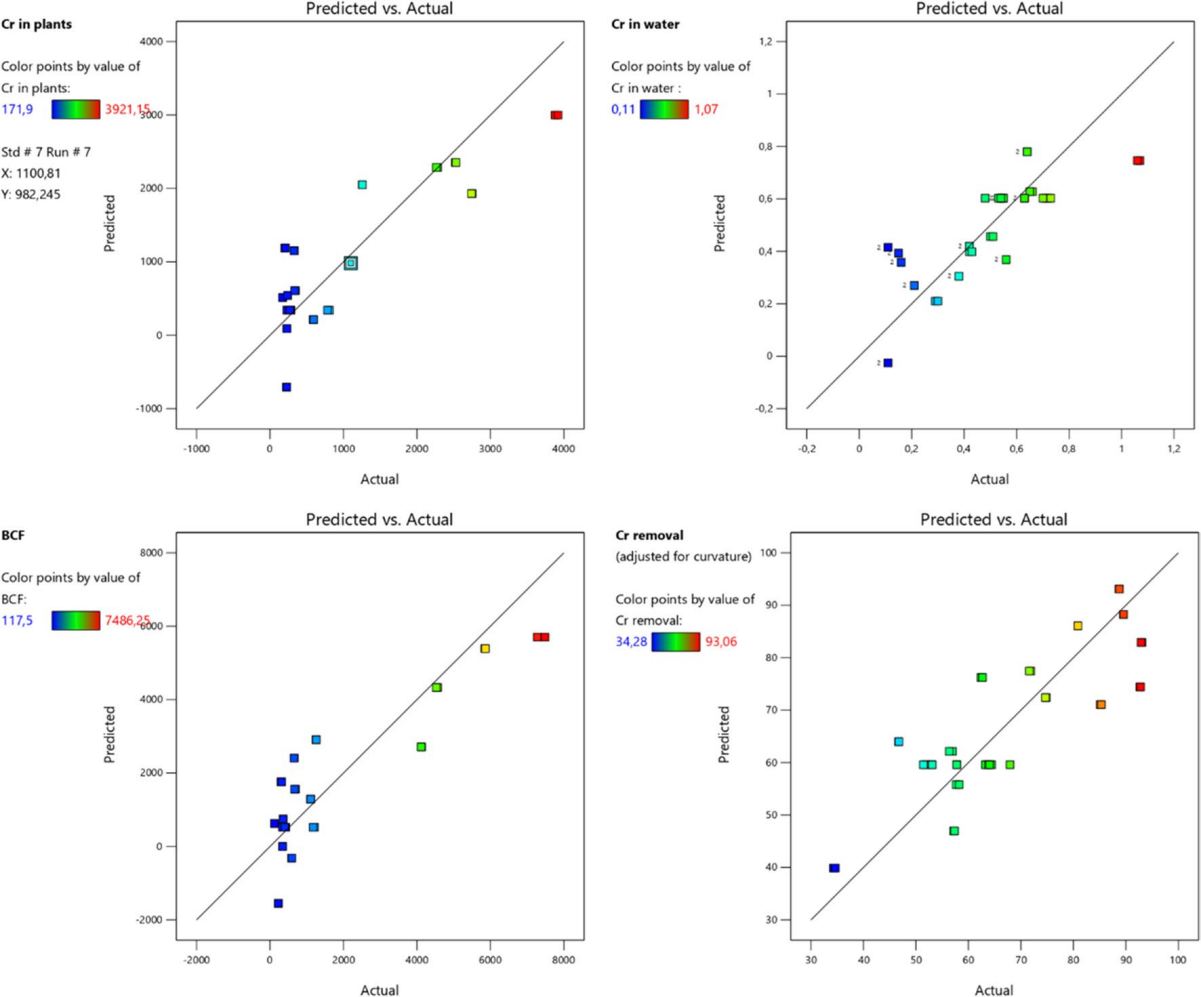
Actual and predicted scores of nano-phytoremediation through RSM

The analysis based on the RF machine learning algorithm validated the results accurately following the actual results due to high *R*^*2*^ scores for all output parameters. The *R*^*2*^ scores were recorded as 0.956 for Cr in water, 0.987 for Cr in plant, 0.992 for BCF, and 0.957 for Cr removal. The results for RMSE and MAE revealed scores of 0.0503 to 185.4536 and 0.0275 to 91.1947, respectively (Table 6). The scores of both performance metrics exhibited the same pattern and were recorded from maximum to minimum as BCF > Cr in plants > Cr removal > Cr in water. A similar pattern was also observed for MAPE and MedAE. The MAPE scores were recorded as 4.916% (Cr in water), 13.541% (Cr in plants), 13.629% (BCF), and 3.187% (Cr removal), whereas the MLSE scores were similar for Cr in plants and BCF (0.071). The MedAE was documented as 0.004 (Cr in water), 10.997 (Cr in plants), 16.990 (BCF), and 0.293 (Cr removal). Relatively very low scores for MLSE were attributed for all output parameters and recorded as 0.001 for Cr in water and 0.003 for Cr removal. Relatively low RRMSE scores were registered for all output factors and ranged between 0.006 and 0.023. The overall performance of output parameters considering all performance metrics showed the lowest scores for Cr in water followed by Cr removal. High scores for all performance metrics are documented for Cr in plant and BCF (Table 6). Figure 6 shows the graphical representation of all outputs’ actual and predicted scores using the firefly-RF algorithm. Due to high *R*^2^ values, all plots show a similar pattern of graphs for all output parameters. Furthermore, the dashed line, also known as the 1:1 line or the identity line, represents a 45° line, which, under ideal conditions, represents perfect predictions. Similarly, a difference between actual and predicted scores acquired from RSM is provided in Fig. 7.

**Table 6:** Performance metrics for the Firefly-RF model

	*R*^*2*^	*RMSE*	*RRMSE (%)*	*MAE*	*MAPE (%)*	*MLSE*	*MedAE*
Cr in Water (mg/L)	0.956	0.050	0.023	0.028	4.92	0.001	0.004
Cr in Plants (mg/Kg)	0.987	66.266	0.019	55.508	13.54	0.071	10.997
BCF	0.992	185.454	0.013	91.195	13.63	0.071	16.990
Cr Removal (%)	0.957	3.359	0.006	1.860	3.19	0.003	0.293

## Discussion

Phytoremediation studies of aquatic plants are highly significant due to the direct exposure of aquatic plants to the pollutant-enriched aquatic bodies. However, the success of phytoremediation is interlinked with a variety of variable factors ranging from plant to the type of pollutant and the addition of different additives to enhance the phytoremediation potential. The coontail (*C. demersum*) is an efficient hyperaccumulator plant used for the phytoremediation of a variety of pollutants ranging from inorganic metals or compounds (Ewadh 2020) to radioactive elements (Markich 2013).

The efficiency of phytoremediation using hyperaccumulator plants relies on the collection of plants from a pollutant-free environment (Terzi and Yıldız, 2011). The studies investigating the phytoremediation of *C. demersum* plants are generally based on collection from natural resources (Abdallah, 2012; Hassan and Al-Khalidi 2018) with a high possibility of already being contaminated with different types of pollutants. The availability of contaminated-free plants for phytoremediation is highly critical, and plants propagated through plant tissue culture offer a novel way of plants availability for phytoremediation studies of *C. demersum* plants in recent years (Aasim et al. 2023c; Dogan et al. 2018). The advantage of using plant tissue cultured plants is the clonal production under a controlled environment without heavy metal exposure from a tiny explant (Karatas et al. 2015).

The success of the phytoremediation model depends on the experimental model used for investigation. Most of the studies on phytoremediation using aquatic plants are based on linear analysis of input variables (Dogan et al. 2018). In recent years, the application of non-linear models like response surface methodology has been employed for optimizing the input factors of phytoremediation studies for more environmental sustainability (Darajeh et al. 2016; Ferreira et al. 2023). The experiment is performed according to the inputs designed by RSM via DOE. The output parameters attained through experiments can be analyzed by RSM in different ways ranging from the validity of the model to optimizing input factors with a given target (Kumar et al. 2018). The use of RSM for optimizing the phytoremediation potential of different plants has been documented in recent years for different types of pollutants (Kasman et al. 2019; Kumar et al. 2018; Li et al. 2021; Mohamad Thani et al. 2020). In the present study, three different input variables were successfully used for optimizing the phytoremediation potential of in vitro-induced *C. demersum* plantlets against Cr from an aqueous medium. Results demonstrated the supremacy of RSM for optimizing input variables and employed for other aquatic plants like Mexican Sword-plant (Kasman et al. 2019), water hyacinth (Kumar et al. 2018), *Alocasia puber* (Hassk.) Schott (Mohamad Thani et al. 2020), and marine alga (*Gelidium amansii* J.V. Lamour) (El-Naggar et al. 2018)***.***

The advantage of using RSM is not only optimization, but also enables checking the overall performance of the model (Kumar et al. 2018). The chance of a larger “Model F-Value” is very rare (0.01%), and generally occurs due to noise. The value of “Prob > F” less than 0.0500 illustrates the significant model and values greater than 0.1000 reflect the not significant model (Kumar et al. 2018; Mohamad Thani et al. 2020). The analysis of response surface regression revealed satisfactory *R*^*2*^ scores for all output parameters. However, relatively low *R*^*2*^ predicted scores were documented (Kasman et al. 2019), which resulted in variable predicted values for all output parameters. It is noticeable from the results that the performance of the model can be analyzed by checking the difference between actual and predicted *R*^*2*^ scores of individual output parameters (Mohamad Thani et al. 2020). Results revealed statistically significant models for all output parameters, which illustrated the precise impact of input variables on all phytoremediation parameters (Kumar et al. 2018). The regression model equation from RSM also provides an opportunity to confirm the results and document for removal of Pb from aqueous solution using a Mexican Sword plant (Kasman et al. 2019)*.*

The type of salt, concentration, amount of samples (g/L plants), and exposure time are some of the other variable factors regulating the whole phytoremediation efficiency (Aasim et al. 2023a; Dogan et al. 2018; Hassan and Al-Khalidi 2018; Markich 2013). Statistically variable impact on all output parameters was documented for individual input variables. A comparison of input variables revealed the better performance of NP. Enrichment of phytoremediation medium with TiO_2_NPs exhibited a statistically significant impact on the phytoremediation potential of *C. demersum*. The TiO_2_NPs are documented to promote plant biomass and are already employed with plant growth-promoting rhizobacteria for successful phytoremediation of Cd from soil by using *Trifolium repens* L. (Zand et al. 2020). Likewise, other studies on plants also revealed the positive impact of TiO_2_NPs on the phytoremediation of antimony from soil (Zand and Heir 2020) and heavy metals like Cs, Au, and As from copper mines (Seifi and Dehghani 2021). The results illustrated the beneficial role of TiO_2_NPs for phytoremediation and more research to understand the biochemical activities and pathways.

A comparison of input variables revealed the better performance of NP × *T* with a statistically significant impact on all output parameters. It was followed by Cr concentration, which affected the Cr in plants, BCF, and Cr removal. The results further illustrated that exposure time alone and *C* × *T* had no impact on phytoremediation. Application of TiO_2_NPs significantly affected the Cr in water only and Cr removal from NP × C. These results clearly illustrated the significance of Cr concentration for phytoremediation and the interaction of NP with exposure time. Results indicated the Cr removal (%) of 90.0% or above from different input combinations using RSM. Previous studies on heavy metal removal from aqueous medium using RSM documented the high removal percentage of Cr(IV) using plant powder (Mojiri et al. 2018), Cd and Pb (Kumar et al. 2018), Ni (Mohamad Thani et al. 2020), and Pb (Kasman et al. 2019).

Pareto and normal plots are potent graphical tools for the investigation of significance level and impact of input factors on output parameters. The system is based on the use of a standardized line and the distribution of input variables on the left and right sides of the line. The variables on the right side and left side of the standardized line of the Pareto chart depict the statistically significant and insignificant impact of input variables, respectively, on their respective output parameters. Conversely, the distribution of input parameters is based on the shape and color, positioning of the input variables around the line, and positioning from the top to the bottom in the normal plots. The red-square and blue-round input variables depict the significant and insignificant impact, respectively. The input variables on the right side reflect the direct proportionally impact of the input variable on its respective output variable, while variables placed on the left side of the line present the inverse proportional relationship between input and output parameters. The positioning of the input variables placed near the line presents a low impact, and contrarily, variables placed far away from the line reflect the greater impact of input variables on their respective output parameters. Another advantage of the normal plot is the placement of input variables which reflects relatively more impact of the variable placed on the top of the table and vice versa low impact for the variable placed at the bottom (Katirci 2015). Investigation of both Pareto charts and normal plots exhibited a clear relationship between input and output parameters. The kind of NPs had little effect on Cr in plants but had a large influence on Cr in water and Cr removal. However, all output variables exhibited the direct proportional impact of all input variables on BCF irrespective of statistically significant and insignificant impact. The use of both Pareto charts and normal plots in plant sciences or phytoremediation is fairly limited irrespective of its significance. The studies on IAA production using the plant growth-promoting ability of *Streptomyces fradiae* (Myo et al. 2019) and biosynthesis of gold nanoparticles using Arabic coffee (Keijok et al. 2019) were based on RSM. The use of normal plots to identify the significant level of input variables for in vitro regeneration of sorghum has been documented recently (Aasim et al. 2023b).

The illustration of data in a different graphical presentation like contour and surface plots makes it attractive to optimize the responses between two input variables (Aasim et al. 2023b, 2023c). In this study, both plots were constructed for NP × *C*, NP × *T*, and Cr × *T*. Distribution of output parameters on the *x*-axis and *y*-axis reflected with different colors exhibited the optimization of input parameter with a desired target (Aasim et al. 2023c; Kasman et al. 2019). The contour plots can be used to find the desired target by checking the input values of both axes. The results were further confirmed by surface plots which distribute the data in 3D format (Ferreira et al. 2023). The use of both contour plots and surface plots have been employed for phytoremediation studies in different plants and pollutants (El-Naggar et al. 2018; Jaskulak et al. 2020; Kasman et al. 2019; Kumar et al. 2018; Mohamad Thani et al. 2020). The response optimizer generated a different combination of input parameters depending on the target set. Response optimizer is a powerful tool employed for optimizing Cd concentration and exposure time for the phytoremediation potential of *C. demersum* (Aasim et al. 2023c). Heatmap is a powerful statistical tool used for finding the linear relationship between input and output parameters. Correlation values vary from − 1 to + 1, and values close to 0 indicate that there does not exist any linear relationship between the two variables. The correlation closer to one reflects the positive or proportional relationship. A correlation of − 1 is similar and exhibits the reciprocal relationship between both variables. The use of heatmaps for AI-based studies has also been documented (Aasim et al. 2023d).

Application of AI-based models for heavy metal removal (Shanmugaprakash et al. 2018; Singh et al. 2022) or phytoremediation (Aasim et al. 2023c) have been used extensively for prediction, validation, and optimization studies. Extensive investigation of heavy metal studies revealed the use of either single or hybrid models (Fan et al. 2017; Shi et al. 2023). However, the use of AI techniques related to phytoremediation is quite low (Titah et al. 2018), especially for aquatic plants (Aasim et al. 2023c). The advantage of employing AI-based models is to optimize the input variables precisely (Razzaghi et al. 2018) compared to traditional algorithms due to certain disadvantages or relative problems (Balasubramani et al. 2020; Shi et al. 2023). The optimization of hyperparameters in AI/ML-based algorithms is highly critical, and recent advancements in AI allow us to use AutoML programs like Firefly to optimize the hyperparameters.

In this study, the Firefly algorithm, in conjunction with the Firefly-RF, was utilized to predict and validate phytoremediation parameters. The effectiveness of this nature-inspired algorithm is particularly noteworthy, demonstrating superior performance compared to grid search in hyperparameter tuning. Its efficiency lies in the ability to rapidly converge to the same solution or a closely related one, making it a potent tool in the optimization process. The Firefly algorithm has been employed to support vector machine parameter tuning (Chao and Horng 2015; Tuba et al. 2016). However, there is no documented report on the use of the Firefly algorithm for phytoremediation studies. On the contrary, the use of the RF model for phytoremediation studies like the use of monkeygrass for the phytoextraction of Zn from soil (Janani et al. 2019) and Cd from aqueous solution using *C. demersum* (Aasim et al. 2023c) for the prediction and validation exist. Our findings showed relatively high *R*^*2*^ scores over 0.95 for all output parameters. The high *R*^*2*^ score near 1.0 reflects the excellent predicted values for all output variables. The results were further confirmed by controlling the other performance metrics, and low scores were attributed to all performance metrics for Cr in water and Cr removal (%). Since all RRMSE (%) values are less than 1.0%, it shows excellent predictive capability of the ML model. The results confirmed that *C. demersum* plants successfully accumulated the Cr, confirmed by a previous study on the same plant (Aasim et al. 2023a, b, c, d). Several studies on aquatic plants like *Azolla pinnata* (R. Br.) also presented the *R*^*2*^ and MSE scores for the removal of malachite green (Kooh et al. 2016). The use of ML modeling for phytoremediation of heavy metals in soils has already been documented (Shi et al. 2023) like immobilization efficiency in biochar-amended soils (Palansooriya et al. 2022), and Cd removal by *Sinapas alba* L. (Jaskulak et al. 2020). The outcomes demonstrate the viability of using AI-based models for phytoremediation study data validation, prediction, and optimization.

## Conclusion

The mainstay of applying technology for commercial purposes lies in the validation and prediction of experiment-derived data. Addressing the challenges of phytoremediation in water bodies, the utilization of aquatic plants like *C. demersum* offers sustainable solutions. Our findings suggest that the efficiency of phytoremediation can be elevated by incorporating NPs for future nano-phytoremediation, targeting diverse pollutants in aquatic systems. It is essential to assess the efficacy of NPs under controlled field conditions for comprehensive phytoremediation studies. The novel Firefly-RF model, with high *R*^2^ values across various phytoremediation parameters, emphasizes the significance of the experiment. This study underscores the promising application of AI in phytoremediation, prompting the need for increased attention, emphasis, and additional studies to validate, enhance, and establish similar innovative protocols.

## Data Availability

The datasets analyzed or generated during the research work are available on reasonable request.

## Footnotes

1 https://github.com/timzatko/Sklearn-Nature-Inspired-Algorithms
